# Detection of Balenine in Mouse Plasma after Administration of Opah-Derived Balenine by HPLC with PITC Pre-Column Derivatization

**DOI:** 10.3390/foods11040590

**Published:** 2022-02-18

**Authors:** Yasutaka Shigemura, Yu Iwasaki, Yoshio Sato, Tomomi Kato, Takuya Seko, Kenji Ishihara

**Affiliations:** 1 Department of Nutrition, Faculty of Domestic Science, Tokyo Kasei University, 1-18-1 Kaga, Itabashi-ku, Tokyo 173-8602, Japan; iwasaki-y@tokyo-kasei.ac.jp (Y.I.); satouy@tokyo-kasei.ac.jp (Y.S.); 2 Seafood Safety and Technology Division, Fisheries Technology Institute, Japan Fisheries Research and Education Agency, 2-12-4 Fukuura, Kanazawa, Yokohama 236-8648, Japan; takasawa@agate.plala.or.jp (T.K.); sekotakuya@affrc.go.jp (T.S.); hplc@affrc.go.jp (K.I.)

**Keywords:** balenine, opah, carnosine, anserine, phenyl isothiocyanate, pre-column derivatization

## Abstract

We examined the absorption of balenine (Bal) in mouse blood after the administration of a high-purity Bal prepared from opah muscle. Using HPLC with phenyl isothiocyanate pre-column derivatization, we successfully isolated imidazole peptides and their constituents. We detected Bal and 3-methylhistidine (3-Me-His) in mouse blood 1 h after the administration of opah-derived Bal. The concentrations of Bal and 3-Me-His significantly increased to 128.27 and 69.09 nmol/mL in plasma, respectively, but were undetectable in control and carnosine (Car)-administrated mice. In contrast, β-alanine and histidine did not increase in mouse plasma 1 h after the administration of Car and opah-derived Bal. The present study is the first report on the absorption of food-derived Bal in mouse blood and serves as a pilot study for future clinical trials.

## 1. Introduction

Balenine (Bal), also known as ophidine, is an imidazole dipeptide that is composed of β-alanine (β-Ala) and 3-methylhistidine (3-Me-His). Bal is not as well known as the major imidazole dipeptides carnosine (Car) and anserine (Ans). Car and Ans contain β-Ala as a counterpart to histidine and 1-methylhistidine, respectively. One reason for the obscurity of Bal might be its low prevalence in nature among the imidazole dipeptides [1]. Car is mainly distributed in the muscles of artiodactyls, such as cattle, pigs, horses, and donkeys, and in other vertebrates, such as humans, sturgeons, eels, and frogs. Ans is mainly distributed in the muscles of birds, such as chickens, turkeys, and rooks, and in fishes, such as marlins, salmons, and croakers. In addition, Car widely coexists with Ans in the muscles of many animals that are food materials, so the biochemistry, molecular biology, pharmacology, neuroscience, and physiology of Car have been widely studied and compared with Ans [2]. Bal has also been detected in the muscles of cetaceans, such as sperm whales, blue whales, and fin whales, and in snakes, such as sea snakes and cobras [1]. In contrast to the few studies on the functionality of Bal, many studies on the antioxidant activities of Car and Ans have been published [3,4,5,6,7,8]. Human trials have demonstrated that supplementation with Car reduces plasma soluble transferrin receptor and serum resistin concentrations in obese subjects [9,10], similar to in vitro studies. In addition, Elbarbary et al. reported that Car supplementation decreases hemoglobin A1c (HbA1c) levels in patients with diabetic nephropathy compared to placebo [11]. Moreover, recent human studies have suggested that supplementation with Car and Ans improves cognitive function in elderly subjects [12,13,14,15,16,17]. Thus, imidazole dipeptides are promising food-derived di-peptides with health benefits.

Wada et al. reported that 26 weeks of Bal supplementation improves learning and memory formation, and it changes brain characteristics in Alzheimer’s disease model mice compared to mice without Bal supplementation [18]. Very recently, Yang et al. demonstrated that superoxide dismutase is activated by the treatment of myotubes with Bal, and superoxide dismutase activity increases in the skeletal muscles of Bal-fed mice [19,20]. Takahashi et al. reported that Bal reduces peptidyl prolyl cis-/trans-isomerase, which is associated with the pathogenesis of cancer, asthma, neurodegenerative diseases, nonalcoholic steatohepatitis, and viral infections [21]. However, because of its distribution in inedible animal muscles, the health functionalities of Bal as a food material have not been fully studied in human trials. Very recently, Bal was detected in the muscles of opah (Lampris guttatus), a mesopelagic and regionally endothermic fish, in high concentrations [22]. Omura et al. detected 18.89 and 25.61 g/kg wet weight of Bal in minke whale and opah, respectively, but none in bigeye tuna and albacore. They also reported that Bal was distributed at higher concentrations than Car and Ans in opah muscle, suggesting that opah is a superior source of food-derived Bal compared to whale muscle. Our previous study also successfully demonstrated the isolation of highly concentrated Bal from opah muscle, and its significantly higher antioxidant and Fe (II) ion-chelating ability compared to Car and Ans [23].

To demonstrate the possible Bal health benefits, which have been demonstrated by in vitro studies using practical amounts, it is necessary to evaluate its bioavailability characteristics, such as its absorption into blood after ingestion. Kubomura et al. reported that the ingestion of 2.0 g Ans/60 kg body weight increased Ans plasma levels by a maximum of 13.4 μg/mL in a human trial [24]. Everaert et al. reported that the ingestion of Ans dose dependently increases Ans concentrations in human plasma and urine [25]. Although these examples of Ans absorption are from human studies after supplementation, Car absorption has been demonstrated in human and pig blood after daily consumption of beef without supplementation [26,27]. Based on previous studies, it has been reported that these imidazole peptides might be transported via a peptide transporter, such as peptide transporter 1 (PEPT1), and hydrolyzed by carnosinase inside enterocytes or in blood circulation [1,28,29].

Based on previous studies, we hypothesized that Bal ingestion increases Bal, β-Ala, and 3-Me-His concentrations in blood with the same mechanisms as those of Ans absorption. To verify the hypothesis, Bal absorption must be elucidated in detail to support its health functionalities as demonstrated by in vitro studies, which may increase resource utilization and the value of opah muscle. In addition, to evaluate Bal as a practical supplement, its absorption must be compared with that of Car, which is abundant in supplements, though the bioavailability of single-Car substances is unresolved. Therefore, the objective of this study was to detect food-derived Bal and its constituent amino acids in blood after the ingestion of opah-derived Bal using mice as a precursor to human trials, and to compare the contents in plasma with those of Car and its constituent amino acids. To detect opah-derived Bal, β-Ala, and 3-Me-His as distinct from other endogenous amino acids and peptides in blood, it is necessary to have high resolution and sensitivity. However, previous studies using high-performance liquid chromatography (HPLC) and liquid chromatography–mass spectrometry (LC-MS) analyses have not fully verified the resolution of these imidazole peptides and their constituents in blood. Therefore, we also attempted to define the HPLC resolution of Bal, β-Ala, and 3-Me-His from Car, Ans, and α-amino acids in blood using several types of columns and amino acid derivatization reagents.

## 2. Materials and Methods

### 2.1. Balenine Samples

**Figure 1:**
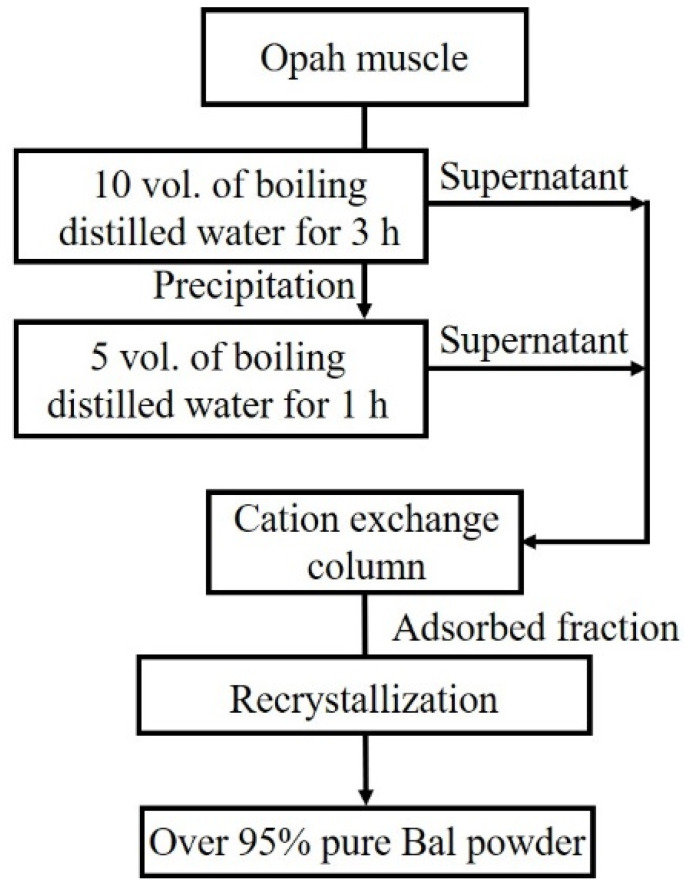
Schematic representation of the study design. Balenine was prepared from opah muscle through boiling, ion-exchange, and recrystallization treatments.

**Figure 2:**
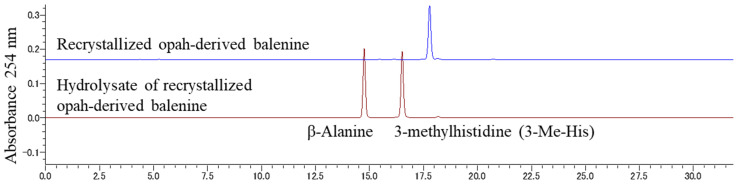
Pre-column derivatization HPLC chromatogram of opah-extracted balenine and hydrolyzed balenine. The chromatograms show balenine and its hydrolysate without impurities.

As shown in Figure 1 Bal was prepared from opah muscles according to our previously reported method as follows [23]: Opah muscle fillet from dorsal ordinary muscle was minced and extracted in boiling water. The water-soluble fraction was recovered after centrifugation and applied to Amberlite IR120BNA cation-exchange beads (Organo Corporation, Tokyo, Japan) packed into a 50 × 300 mm Econo-pac chromatography column (Bio-Rad Laboratory, Hercules, CA, USA). The adsorbed fraction was eluted with 20 mM NaOH, and the cation-exchange-absorbed fractions were lyophilized. The lyophilized powder was dissolved in 70 °C preheated ethanol. After 5 min of stirring, water was added, and the solution was stirred for 1 h. The ethanol-insoluble fraction was removed using a GF/C glass filter (GE Healthcare, Tokyo, Japan), and ethanol was added to the filtered solution. The solution was cooled to −20 °C for 3 h, and then isopropanol was added. After cooling the solution at −20 °C overnight, precipitated Bal crystals were collected by filtration using a GF/C glass filter. The crystals were dried under a vacuum overnight and used as purified Bal (Figure 1). The purity of the recovered Bal was estimated by phenyl isothiocyanate (PITC) pre-column derivatization HPLC (Figure 2). Over 95% of purified Bal was used for supplementation for mice.

### 2.2. Chemicals

A standard mixture of amino acids (type H), other amino acids, and imidazole peptides (such as L-hydroxyproline, L-asparagine, L-glutamine, β-Ala, L-Car, and L-Ans) were purchased from FUJIFILM Wako Pure Chemical Corporation (Osaka, Japan). Bal was purchased from Hamari Chemicals (Osaka, Japan). Other reagents, including ammonium acetate, acetonitrile (HPLC grade), trifluoroacetic acid (TFA), and PITC, were purchased from FUJIFILM Wako Pure Chemical Corporation. Triethylamine was purchased from Thermo Fisher Scientific (Life Technologies, Carlsbad, CA, USA).

### 2.3. Resolution of Balenine and Related Amino Acids by HPLC

To define the conditions for HPLC resolution of Bal and constituent amino acids from α-amino acids, standard reagents were used in two HPLC conditions. PITC pre-column derivatization for HPLC resolution was performed using the method of Bidlingmeyer et al. [30] with slight modification [31,32]. Bal, Car, Ans, and amino acids were derivatized with PITC, and the resultant phenylthiocarbamyl (PTC) derivatives were resolved on a Kinetex EVO C18 column (5 μm, 4.6 mm × 250 mm, Phenomenex, Torrance, CA, USA). Binary gradient elution was performed with 0.01% TFA (solvent A) and 60% acetonitrile (solvent B) at a flow rate of 0.5 mL/min. The column was equilibrated with 15% B. The gradient profile was as follows: 0–30 min, 15–75% B; 30–35 min, 75–100% B; 35–40 min, 100% B; 40–40.1 min, 100–15% B; 40.1–50 min, 15% B. The column was maintained at 45 °C, and the absorbance at 254 nm was monitored.

The PTC derivatives of standard reagents were resolved on an Inertsil ODS-3 column (5 μm, 4.6 mm × 250 mm, GL Science, Tokyo, Japan) using an HPLC system (Shimadzu, Kyoto Japan). Binary gradient elution was performed with 150 mM ammonium acetate containing 5% acetonitrile pH 6.0 (solvent A) and 60% acetonitrile (solvent B) at a flow rate of 0.5 mL/min. The column was equilibrated with 100% solvent A. The gradient profile was as follows: 0–0.1 min, 0% B; 0.1–1.01 min, 0–10% B; 1.01–20 min, 10–47.5% B; 20–25 min, 47.5–100% B; 25–37 min, 100% B; 37–37.1 min 100–0% B; 37.1–50 min, 0% B. The column was maintained at 45 °C, and the absorbance at 254 nm was monitored.

### 2.4. Administration of Imidazole Peptides and Blood Collection from Mice

Blood collection from mice after administration of samples was conducted according to the Helsinki Declaration, and all procedures were approved by the Animal Experimentation Committee of the Fisheries Technology Institute, Japan Fisheries Research and Education Agency, code H30-1. Four-week-old ddY male mice were purchased from Japan SLC (Hamamatsu, Shizuoka, Japan) and fed for 12 weeks. Nine of these 16-week-old mice were divided into three groups. Each group was administrated either 200 mg/kg body weight of water, Car, or opah-derived Bal with a feeding needle after fasting for 12 h. The body weights of water, Car, and opah-derived Bal groups were 45.87 g ± 4.00, 48.77 g ± 7.38, and 48.80 g ± 2.58, respectively. After 1 h of administration, mice were anesthetized by isoflurane inhalation, and whole blood was collected from the inferior vena cava with a syringe containing sodium heparin as anticoagulant. Plasma was collected after centrifuging at 2500× *g* for 15 min. Three volumes of ethanol were added to the plasma to remove protein by centrifugation (3000× *g*, 10 min). The obtained supernatants, the ethanol-soluble plasma fractions, were then used for HPLC determination of Bal, Car, Ans, β-Ala, 3-Me-His, and amino acid.

### 2.5. Amino Acid Analysis of Mouse Plasma

The PTC derivatives of mouse plasma samples and standard reagents were resolved by using Kinetex EVO C18 column, 150 mM ammonium acetate containing 5% acetonitrile pH 6.0 (solvent A), and 60% acetonitrile (solvent B). The column was equilibrated with 97% solvent A. The gradient profile was as follows: 0–5 min, 3% B; 5–7.5 min, 3–4% B; 7.5–14 min, 4–8% B; 14–20 min, 8–45% B; 20–30 min, 45–65% B; 30–30.01 min, 65–100% B; 30.01–35 min, 100% B; 37–37.1 min 100–0% B; 35.01–45 min, 3% B. All other HPLC conditions were the same as those described above in Section 2.3.

### 2.6. Statistical Analysis

The differences between the means were evaluated by analysis of variance followed by Tukey’s multiple comparison test (*p* < 0.05) using Excel-Toukei 2010 (Social Survey Research Information Co., Ltd., Tokyo, Japan).

## 3. Results

### 3.1. HPLC Conditions for the Resolution of Bal, β-Ala, and 3-Me-His

**Figure 3:**
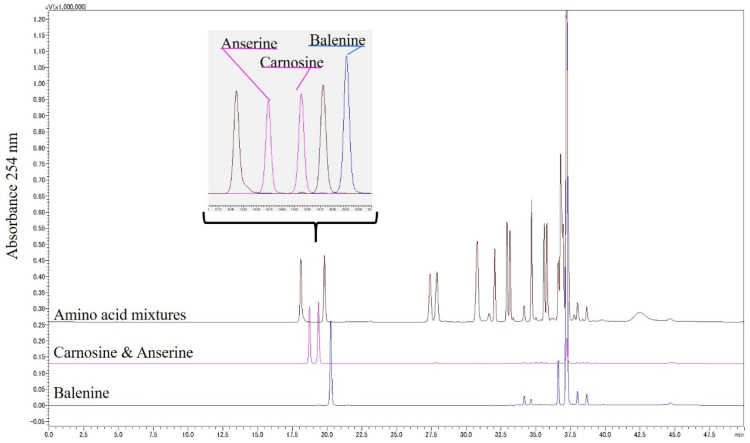
HPLC chromatogram obtained after resolving a PITC-derivatized anserine, carnosine, balenine, and amino acid mixture. The resolution was conducted using a Kinetex EVO C18 column and binary gradient elution with 0.01% trifluoroacetic acid and 60% acetonitrile at a flow rate of 0.5 mL/min.

**Figure 4:**
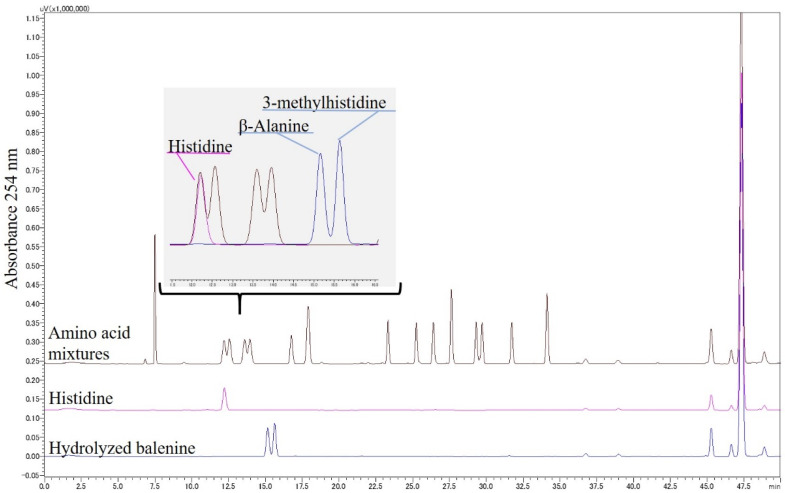
HPLC chromatogram obtained after resolving PITC-derivatized histidine, β-alanine, 3-methylhistidine, and amino acid mixture. The resolution was conducted using an Inertsil ODS-3 column, and binary gradient elution was performed with 150 mM ammonium acetate containing 5% acetonitrile pH 6.0 and 60% acetonitrile at a flow rate of 0.5 mL/min.

Several combinations of reverse-phase columns, solvents, and derivatization reagents were used for the HPLC resolution of Bal, β-Ala, and 3-Me-His from other imidazole peptides and amino acids. The best resolution condition for Bal, Car, and Ans was a combination of PITC derivatization with a Kinetex EVO C18 column, 0.01% TFA, and 60% acetonitrile (details are in Section 2.3). As shown in Figure 3, these imidazole peptides were successively isolated from 17 amino acids, which exist in blood, although it was difficult to isolate β-Ala and 3-Me-His in single peaks. We then found the best resolution conditions for β-Ala and 3-Me-His through trial and error. The optimal condition was a combination of PITC derivatization with an Inertsil ODS-3 column, 150 mM ammonium acetate containing 5% acetonitrile pH 6.0, and 60% acetonitrile (details are in Section 2.3). Both Bal constituent amino acids, β-Ala and 3-Me-His, were prepared by Bal hydrolyzation at 150 °C with 6 M HCl under decompression. Both amino acids were successively isolated from the other amino acids (Figure 4).

### 3.2. HPLC Detection of Food-Derived Bal, Car, and Constituent Amino Acids from Mouse Plasma

**Figure 5:**
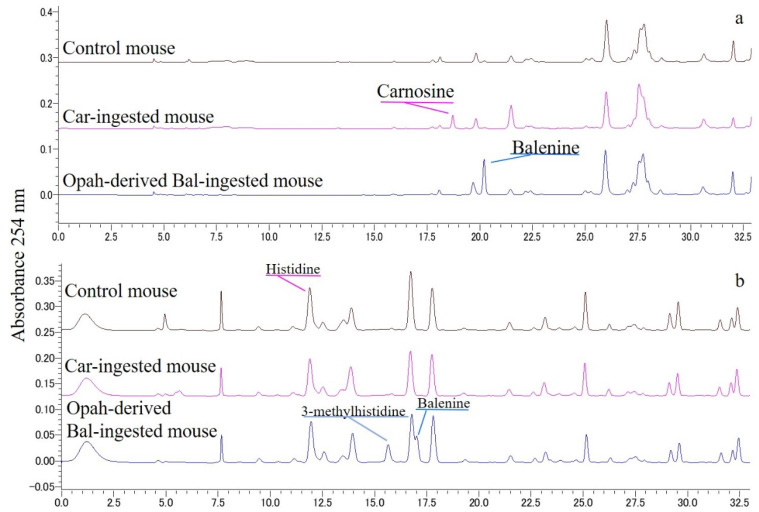
HPLC chromatogram of PITC-derivatized mouse plasma after administration of water (control), carnosine (Car-administrated mouse), and opah-derived balenine (opah-derived Bal-administrated mouse). The chromatogram upper column (**a**) shows detection of carnosine and balenine from mouse plasma 1 h after administration. The chromatogram upper column (**b**) shows the detection of histidine and 3-methylhistidine in mouse plasma 1 h after administration.

As shown in Figure 5, two different HPLC conditions resolved mouse plasmas that were prepared 1 h after the administration of water, Car, and opah-derived Bal. The peaks of Car and Bal were observed only in plasmas after the administration of Car and opah-derived Bal, respectively (Figure 5a), while these peaks were absent in the plasma of water-administrated mice (controls). The Bal constituent 3-Me-His was observed in plasma only after the administration of opah-derived Bal (Figure 5b). However, none of the chromatograms of any sample showed a β-Ala peak, which is a standard reagent that was eluted before the 3-Me-His peak (Figure 4).

### 3.3. Quantitative Comparison of Imidazole Peptides and Amino Acids in Mouse Plasma

**Figure 6:**
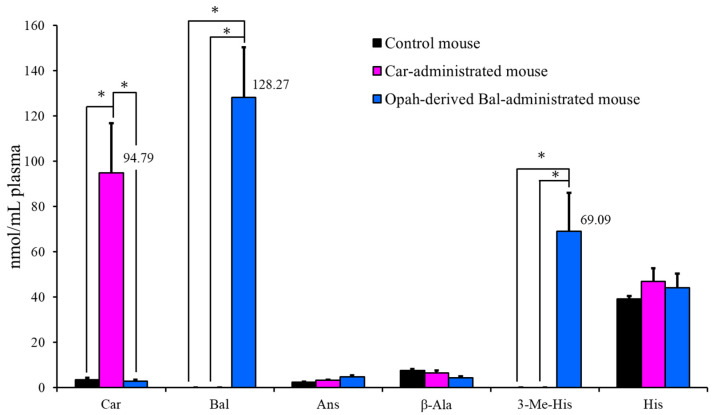
The concentration of imidazole peptides and constituents in mouse plasma 1 h after the administration of water (control), carnosine (Car-administrated mouse), and opah-derived balenine (opah-derived Bal-administrated mouse) at 200 mg/kg body weight. The concentrations of carnosine (Car), balenine (Bal), anserine (Ans), β-alanine (β-Ala), 3-methylhistidine (3-Me-His), and histidine (His) are presented as means ± SD (*n* = 3). Asterisks indicate significant differences (* *p* < 0.01).

Figure 6 shows a quantitative comparison of imidazole peptides and constituents in mouse plasma 1 h after water and imidazole peptide administration. The opah-derived Bal administration increased the levels to 128.27 mol/mL of Bal and 69.09 mol/mL of 3-Me-His in plasma after 1 h. These levels were significantly higher than those in Car-administrated and control mice. Compared with opah-derived Bal-administrated and control mice, the concentration of Car was 94.79 mol/mL in the plasma of Car-administrated mice, which was significantly higher. The increased concentrations of Car and Bal in plasma did not show significant differences with each other. In addition, the concentrations of Ans, His, and β-Ala in plasma in each mouse group did not show significant differences.

## 4. Discussion

**Figure 7:**
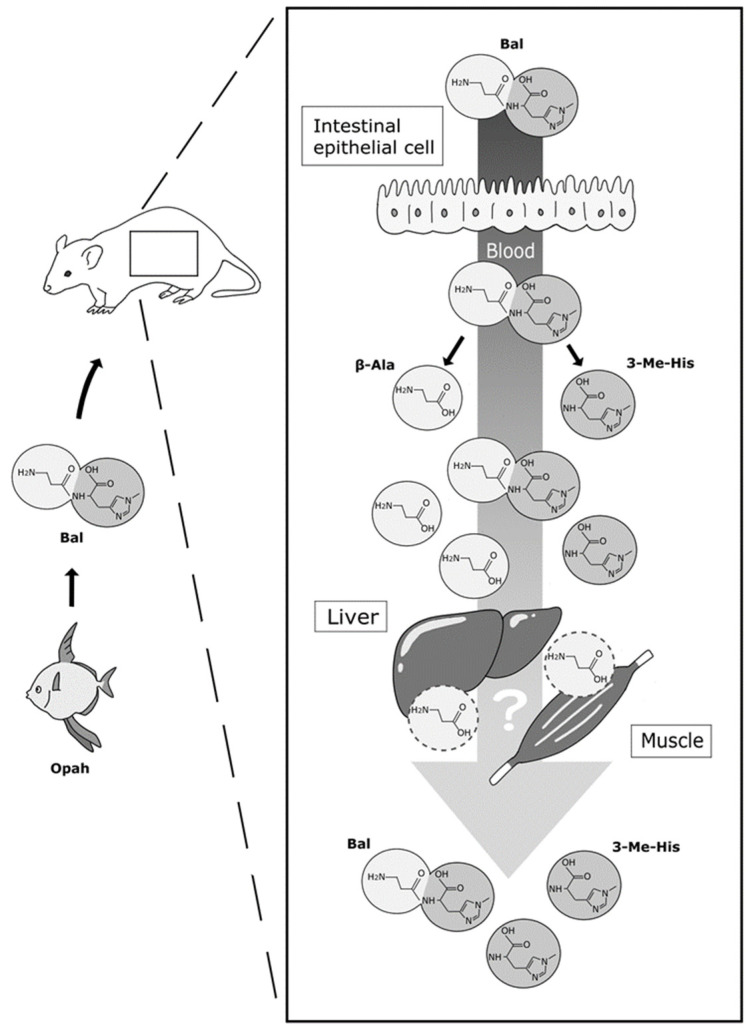
Image of suspected mechanisms for the absorption of Bal and its constituents. Dark and light shades of grey indicate β-Ala and 3-Me-His.

The present study is the first detection of food-derived Bal and its constituent amino acids from mouse blood and serves as a pilot study for human trials. We successfully demonstrated the absorption of Bal and 3-Me-His into mouse blood after the administration of opah-derived Bal. Previous studies that detected Car and Ans from blood, muscle, and urine used HPLC and LC-MS/MS [24,25,26,27,33]. However, these biological samples contained endogenous amino acids. Therefore, it is crucial to verify the resolution conditions to isolate each imidazole peptide distinctly from the endogenous amino acids during the HPLC detection of food-derived imidazole peptides from blood. Although LC-MS analysis may eliminate the risk of insufficient isolation or overlapping peaks of these components, isolation by HPLC is essential due to the common constituent β-Ala and the identical molecular weight of Ans and Bal. Thus, in the present study, we isolated them using pre-column derivatization reagents, such as PITC, carbazole-9-carbonyl chloride, and 6-aminoquinolyl-N-hydroxysuccinimidyl carbamate, in addition to a series of octadecylsilyl (C18), octylsilyl (C8), and pentafluorophenol (PFP) columns. Unfortunately, imidazole dipeptides and amino acids could not be isolated as single peaks by a single HPLC condition. Finally, the two best resolution conditions were obtained using a combination of PITC derivatization with a Kinetex EVO C18 column, and PITC derivatization with an Inertsil ODS-3 column (Figure 4 and Figure 5). As shown in Figure 7, these conditions could be applied to the isolation of Car, Bal, His, β-Ala, 3-Me-His, and His from blood samples to identify imidazole peptides and the absorption of constituents. However, although it was difficult to isolate each imidazole peptide by LC-MS, a more accurate evaluation for the absorption of imidazole peptides in blood must be conducted considering the matrix interference caused by using LC-MS/MS and stable isotope-labeled imidazole peptides. Although the bioactivities of Bal have not been fully reported compared to the other two major imidazole peptides, the present verification of food-derived Bal absorption into mouse blood suggests that the administration of opah-derived Bal has possible health benefits as previously reported using cell culture, animal, and in vitro studies [18,19,20,21,23].

As shown in Figure 6, though the Bal concentration in plasma was 1.3-fold higher than that of Car after administration, there were no significant differences in the absorption of Bal and Car in mouse blood. Thus, administrated Bal might be absorbed into the blood via PEPT1, expressed in mouse small intestine, similar to Car. In addition, the lower Car concentration in blood could be due to Car digestion by carnosinase in blood over the course of 1 h. Kubomura et al. demonstrated that the absorption of food-derived Ans in human blood and the time to maximum concentration (tmax) is 0.52 ± 0.07 h after the ingestion of skipjack tuna muscle-derived Ans. They also reported a significant increase in the Ans constituent 1-methylhistidine (1-Me-His) in human blood after ingestion, though they did not estimate the β-Ala concentration in human blood. They revealed the tmax of 1-Me-His to be 1.19 ± 0.07 h, and the concentration remained high in blood within 240 h from ingestion. Similar results were observed in the present study, in that significantly higher values of Bal and 3-Me-His were detected in blood compared to control mice after 1 h from administration. A constituent amino acid of Bal, 3-Me-His, is a metabolite of muscle breakdown that cannot be reutilized for protein synthesis and is consequently excreted in urine. Therefore, it can be assumed that 3-Me-His, as an end product, cannot be utilized in organs after absorption and is circulated in blood for a few hours after administration. However, its counterpart, β-Ala, is not detected in mouse blood after ingestion. Stautemas et al. reported increases in β-Ala in human blood a few hours after its ingestion, and this suggests that β-Ala can be absorbed into the blood from the small intestine [34]. The imidazole peptide samples used in the present administration test do not contain free β-Ala and 3-Me-His. Thus, a part of food-derived Bal and Car could be degraded either in the digestive tract or in other organs after absorption, and degradants β-Ala and His were possibly consumed in organs, such as the liver and muscle, within 1 h after administration, while 3-Me-His was not consumed (Figure 7). However, as seen in the free amino acid compositions (Table 1), the administration of Bal and Car did not affect endogenous amino acid concentrations in the blood, which suggests that the metabolism of imidazole peptides and constituent amino acids did not affect the degradation of proteins or the synthesis of free amino acids.

These conjectures might be tested by detecting food-derived Bal and its constituents in blood shortly after ingestion. Additionally, the present study revealed the absorption of food-derived Bal in mouse blood, which serves as a pilot study for future human trials. Thus, further studies will examine opah-derived Bal in human blood after the ingestion of opah meat extracts. The present results and further studies may contribute to the development of novel functional food components and the utilization of an underused resource by adding value to opah meat.

## 5. Conclusions

In the present study, we successfully isolated imidazole peptides from amino acids using two different HPLC conditions with a PITC pre-column derivatization method. The procedures were applied for the isolation of Car and Bal in mouse blood after the administration of Car and opah-derived Bal, respectively. Both imidazole peptides were significantly increased in blood after administration compared to the control, and both peptides were equally absorbed. However, the constituents of these peptides, β-Ala and His, did not show significant increases, while 3-Me-His remained significantly high in plasma. These results partially confirm our hypothesis that Bal ingestion can increase food-derived Bal and 3-Me-His concentrations in blood. In addition, the results indicate the potential health benefits of opah-derived Bal, which have previously been reported in cell culture, animal, and in vitro studies. Future studies, such as human trials, and the application of a more accurate quantification method are required to demonstrate the absorption and bioactivities of opah-derived Bal, and they are now in progress.

## Figures and Tables

**Table 1:** Amino acid concentrations in mouse plasma after 1 h from administration of water (control) carnosine (Car) and opah-derived balenine (Bal).

nmol/mL Plasma	Control	Car-Administrated Mouse	Bal-Administrated Mouse
Asp	30.46 ± 2.15	30.56 ± 9.69	34.84 ± 1.89
Glu	64.13 ± 7.80	59.20 ± 24.31	67.62 ± 10.77
Asn	16.94 ± 3.79	14.19 ± 4.28	18.64 ± 3.45
Ser	84.95 ± 15.22	70.14 ± 6.08	95.44 ± 4.15
Gln	224.71 ± 6.42	205.01 ± 33.75	276.79 ± 6.16
Gly	179.38 ± 8.94	154.14 ± 18.80	193.63 ± 36.79
His	39.20 ± 1.36	47.02 ± 5.75	44.08 ± 6.36
Arg	53.21 ± 25.01	43.13 ± 22.16	31.66 ± 3.48
Thr	94.71 ± 12.98	73.47 ± 9.67	90.78 ± 4.61
Ala	282.50 ± 46.88	235.93 ± 38.22	303.85 ± 22.53
Pro	101.35 ± 10.30	96.48 ± 12.92	111.30 ± 11.03
Tyr	92.53 ± 10.24	81.38 ± 17.60	90.61 ± 9.93
Val	218.21 ± 9.05	193.44 ± 36.32	228.41 ± 22.89
Met	41.92 ± 3.43	35.91 ± 3.97	42.74 ± 2.64
Cys	15.78 ± 3.54	22.80 ± 7.87	22.71 ± 3.65
Ile	90.94 ± 5.93	76.78 ± 18.73	94.37 ± 12.55
Leu	120.52 ± 15.26	106.71 ± 22.03	129.08 ± 14.32
Phe	56.86 ± 4.36	54.01 ± 7.51	64.03 ± 5.93
Lys	100.91 ± 4.09	93.03 ± 5.14	96.67 ± 4.82

No significant differences were observed in each amino acid among control, Car, and Bal.
